# Letter to the Editor: Oral risedronate increases Gruen zone bone mineral density after primary total hip arthroplasty: a meta-analysis

**DOI:** 10.1186/s13018-021-02891-8

**Published:** 2022-01-05

**Authors:** Kai Huang, Gang Wang, Yi Zeng

**Affiliations:** 1 Department of Orthopaedics and National Clinical Research Center for Geriatrics, Orthopaedic Research Institute, West China Hospital, Sichuan University, 37 Guoxue Road, Chengdu, Sichuan Province 610041 People’s Republic of China; 2 Laboratory of Stem Cell and Tissue Engineering, Orthopaedic Research Institute, West China Hospital, Sichuan University, Chengdu, 610041 People’s Republic of China

**Dear editors,**

Recently, we read a meta-analysis by Li et al. [1] entitled “Oral risedronate increases Gruen zone bone mineral density after primary total hip arthroplasty: a meta-analysis” with great interest. Periprosthetic bone loss after total hip arthroplasty is an inevitable phenomenon mainly due to stress shielding, which may predispose to aseptic loosening, periprosthetic fractures and challenges at revision surgery. We appreciate the authors’ work in this field, however, some issues in the article that may nullify the conclusion need to be mentioned.

Firstly, the authors declared that they had systematically retrieved electronic databases including PubMed, Embase, Web of Science, Cochrane Library, and Chinese Wanfang database. However, to our knowledge, a study by Yin et al. [2] in Wanfang database was eligible on the basis of inclusion criteria, which could be involved in this meta-analysis and beneficial to draw a more comprehensive and convincing conclusion.

Secondly, we noticed that four of the included studies with short-term follow-up (6–12 months) revealed significant efficacy of risedronate while the left one with relatively longer follow-up (4 years) drew the exact opposite conclusion. Although these studies showed excellent homogeneousness, it was improper to ignore the potential reasons for such difference and simply put them into pooled analysis, which without any doubt would bring extra bias and lead to an incorrect conclusion.

Thirdly, it is obvious that two of the included studies (Skoldenberg, 2011 and Kumar, 2011) were the same article. What’s more, both of them and Muren et al. [3] came from the same clinical cohort. Thus, extracting duplicate data from these three articles for analysis would be more likely to lead to an incorrect conclusion and misleading clinical practice. Given that four of the eligible RCTs were followed up no more than 1 year except Muren et al. (4-year follow-up), we recommend to rule out Muren et al. and conduct a short-term (≤ 1 year) meta-analysis in a reference of the work by Shi et al. [4]. Details of these eligible studies are shown in Table 1.

**Table 1:** Baseline characteristic of four eligible studies not include Muren et al. and Kumar et al. in the meta-analysis

Studies	Year	Study design	Country	No. of patients	Mean age	Female patients	Interventon		Follow up
				(E/P)	(E/P)	(E/P)	Experimental	Placebo	
Kinov	2005	RCT	Bulgaria	12/12	58.3/56	8/7	35 mg risedronate	Placebo	6 months
Yamasaki	2006	RCT	Japan	19/21	66.8/66.7	17/19	2.5 mg/d risedronate	Placebo	6 months
Skoldenberg	2011	RCT	Sweden	36/37	61.2/60.3	22/21	35 mg risedronate	Placebo	1 year
Yin	2013	RCT	China	13/13	61.5/63.7	5/6	5 mg/d risedronate	Placebo	6 months

RCT: Randomized controlled trial, E: Experimental, P: Placebo

Fourthly, we find that there are another four similar meta-analyses published online in 2018 [5–8]. All of them were performed following the guideline of PRISMA and four declared that they were the first meta-analysis on this topic, while none of them had a protocol registration in any platform, such as the Cochrane Library and PROSPERO. The meta-analysis registration is very essential to not only improve the quality of reporting, but also provide transparency and avoid repetitive publications.

Finally, it is not rigorous and persuasive enough for authors to conclude that risedronate could significantly reduce periprosthetic bone loss around an uncemented femoral stem, for the long-standing drug efficacy on periprosthetic bone loss and the benefit of final prognosis are still inconclusive. Larger clinical trials that focus on clinically relevant endpoints with a longer duration of follow-up are warranted.

## Data Availability

Not applicable.

## Abbreviations

RCT: Randomized Controlled Trial
PRISMA: Preferred Reporting Items for Systematic Reviews and Meta-Analyses
